# Pneumokokken-Impfung bei Personen ab 60 Jahren: Zusammenhänge zwischen Impfintention, Wissen und psychologischen Gründen für die Impfentscheidung

**DOI:** 10.1007/s00103-025-04012-w

**Published:** 2025-02-27

**Authors:** Hannah Nordmann, Sarah Anna Katharina Uthoff, Anna Zinkevich, Julia Iwen, Marc Biedermann, Lena Ansmann

**Affiliations:** 1 Department für Versorgungsforschung, Abteilung Präventions- und Rehabilitationsforschung, Carl von Ossietzky Universität Oldenburg, Ammerländer Heerstraße 114–118, 26129 Oldenburg, Deutschland; 2 Medizinische Fakultät und Uniklinik Köln, Institut für Medizinsoziologie, Versorgungsforschung und Rehabilitationswissenschaft (IMVR), Lehrstuhl für Medizinsoziologie, Universität zu Köln, Köln, Deutschland; 3 Abteilung Ambulante Versorgung, Verband der Ersatzkassen e. V., Berlin, Deutschland; 4 Dezernat Sicherstellung und Versorgungsstruktur, Abteilung Versorgungsstruktur, Kassenärztliche Bundesvereinigung, Berlin, Deutschland

**Keywords:** 5C-Skala, Theory of Planned Behavior, Impfmüdigkeit, Risikowahrnehmung, Hausarztpraxen, 5C scale, Theory of planned behavior, Vaccine hesitancy, Risk perception, General practitioner practices

## Abstract

**Einleitung:**

Weltweit stellen Pneumokokken die häufigste Ursache für Morbidität und Mortalität bei Infektionen der unteren Atemwege dar. Trotz Empfehlungen der Ständigen Impfkommission (STIKO) bleibt die Impfquote gegen Pneumokokken bei über 60-Jährigen in Deutschland niedrig. Daher wurden für diese Gruppe die Zusammenhänge zwischen psychologischen Gründen des (Nicht‑)Impfens, Wissen über Pneumokokken, Alter und Geschlecht, früheren Impfentscheidungen und der Pneumokokken-Impfintention untersucht.

**Methoden:**

Die Analyse basiert auf Daten der Studie „ALtersspezifische Impfinanspruchnahme VErbessern“, bei der 2022 1117 Patient:innen ab 60 Jahren befragt wurden. Neben der deskriptiven Auswertung der Querschnittsdaten wurde eine Spearman-Korrelationsanalyse durchgeführt. Zudem wurden in einer linearen Regressionsanalyse prädiktive Variablen identifiziert.

**Ergebnisse:**

Vertrauen in die Sicherheit von Impfungen (β = 0,514, *p* < 0,001) und eine Influenza-Impfung (β = 0,153, *p* < 0,001) in der letzten Saison sind die stärksten Prädiktoren für die Pneumokokken-Impfintention. Zudem zeigt die Einschätzung des von einer Pneumokokken-Infektion ausgehenden Risikos (β = 0,086, *p* = 0,002) einen signifikanten positiven Zusammenhang zur Impfintention. Die durchgeführte Regressionsanalyse konnte 48,8 % der Varianz der Impfintention erklären (R^2^ = 0,488).

**Diskussion:**

Vertrauen und Risikowahrnehmung scheinen entscheidende Faktoren für die Impfentscheidung zu sein. Maßnahmen, die diese Aspekte adressieren, könnten die Impfintention erhöhen. Eine gleichzeitige Impfung gegen Influenza und Pneumokokken könnte sinnvoll sein, da das Erhalten einer Influenza-Impfung in der letzten Saison einen positiven Effekt auf die Pneumokokken-Impfintention zeigte.

## Abstract (en)

**Introduction:**

Worldwide, pneumococci are the most common cause of morbidity and mortality from lower respiratory tract infections. Despite recommendations of the Standing Committee on Vaccination (STIKO), the vaccination rate against pneumococci in over 60-year-olds in Germany remains low. This study therefore investigates the associations between psychological reasons for (non-) vaccination, knowledge about pneumococci, age and gender, previous vaccination decisions, and pneumococcal vaccination intention in over 60-year-olds.

**Methods:**

The analysis is based on data from the study “ALtersspezifische Impfinanspruchnahme VErbessern,” in which 1117 patients aged 60 and over were surveyed in 2022. In addition to the descriptive evaluation of the cross-sectional data, a Spearman correlation analysis was carried out. Predictive variables were also identified in a linear regression analysis.

**Results:**

Confidence in the safety of vaccination (β = 0.514, *p* < 0.001) and receiving the influenza vaccination (β = 0.153, *p* < 0.001) in the last season are the strongest predictors of pneumococcal vaccination intention. In addition, the perceived risk of pneumococcal infection (β = 0.086, *p* = 0.002) showed a significant positive correlation with vaccination intention. The regression analysis performed was able to explain 48.8% of the variance in vaccination intention (R^2^ = 0.488).

**Discussion:**

Confidence and risk perception appear to be decisive factors in the decision to get vaccinated. Measures that address these aspects could increase vaccination intention. A joint administration of the influenza and pneumococcal vaccination could be useful, as receiving an influenza vaccination in the previous season showed a positive effect on the intention to get the pneumococcal vaccination.

## Hintergrund

Weltweit stellen Pneumokokken die häufigste Ursache für Morbidität und Mortalität bei Infektionen der unteren Atemwege dar [1]. Das Bakterium *Streptococcus pneumoniae *verursacht bei Erwachsenen am häufigsten ambulant erworbene Pneumonien, seltener Mittelohrentzündungen und Nasennebenhöhlenentzündungen. Bei invasiven Pneumokokken-Erkrankungen (IPD) dringen die Bakterien in sterile Körperbereiche ein; dies ist bei Bakteriämien, Sepsis und Meningitis der Fall [2]. Anfang 2020 wurde eine bundesweite Meldepflicht für IPD eingeführt, deren Umsetzung durch die COVID-19-Pandemie verzögert wurde, so sind bisher nur unvollständige Daten über Inzidenzen verfügbar. Krankenhausdiagnosestatistiken des Statistischen Bundesamtes zeigen eine Inzidenz von Hospitalisierungen aufgrund von Pneumokokken-Pneumonien von 3,2 pro 100.000 Einwohner:innen in den Jahren 2007 bis 2019 [3]. Für die Pneumokokken-Sepsis lag diese bei 2,9 pro 100.000. Die höchsten Inzidenzen für IPD zeigen sich in den Altersgruppen unter 2 und über 60 Jahren. Deswegen wird diesen Altersgruppen von der Ständigen Impfkommission (STIKO) ein Impfschutz gegen Pneumokokken-Erkrankungen empfohlen, ebenso Personen mit Vorerkrankungen und mit beruflicher Exposition mit Metalldämpfen [3]. Auswertungen von Abrechnungsdaten der Kassenärztlichen Vereinigung Bayerns (KVB) zeigen eine Impfquote von 20,1 % der über 60-Jährigen im Jahr 2021, während sie 2017 noch bei 2,4 % lag [4]. Deutschlandweit waren im ersten Quartal 2022 über 40 % der 74-Jährigen gegen Pneumokokken geimpft [5] und auch die Daten der KVB zeigen die höchste Impfinanspruchnahme in der Gruppe der 70- bis 79-Jährigen [4]. Die Impfquote ist bei den über 60-Jährigen in den Altersgruppen 60–69 Jahre und über 90 Jahre am geringsten. Trotz der STIKO-Empfehlung und der möglichen Folgen einer Pneumokokken-Erkrankung ist die Impfinanspruchnahme bei Personen ab 60 Jahren in Deutschland insgesamt gering. Die unzureichende Impfinanspruchnahme kann eventuell durch eine Impfmüdigkeit erklärt werden.

### Vaccine Hesitancy: Das Phänomen der Impfmüdigkeit

Impfmüdigkeit beschreibt den Verzicht auf eine Impfung, obwohl diese verfügbar, sicher und effektiv ist [6]. Impfmüdigkeit ist ein komplexes, kontextspezifisches Phänomen, das von Faktoren wie Risikowahrnehmung, Bequemlichkeit und Vertrauen in die Impfung sowie in das Gesundheitssystem beeinflusst wird [6]. Sie beschreibt das Spektrum zwischen vollständiger Akzeptanz und kategorischer Ablehnung von Impfungen. Das Verständnis der Ursachen der Impfmüdigkeit kann zur Entwicklung von Maßnahmen zur Erhöhung von Impfquoten beitragen. Die 5C-Skala wurde entwickelt, um psychologische Gründe des (Nicht‑)Impfens, die sich auf die Impfmüdigkeit auswirken, zu erfassen und Impfverhalten besser erklären zu können [7].

### Die 5C-Skala

Die 5C-Skala [8] erklärt Impfverhalten durch 5 psychologische Konstrukte:

1. Vertrauen (Confidence) in die Sicherheit und Effektivität der Impfung sowie in das Gesundheitssystem erhöht die Impfbereitschaft,
2. eine niedrige Risikowahrnehmung (Complacency) senkt dagegen die wahrgenommene Notwendigkeit einer Impfung,
3. Barrieren (Constraints), wie physische oder soziale Barrieren, verringern ebenfalls die Impfbereitschaft,
4. Risiko-Nutzen-Abwägung (Calculation) beschreibt das Ausmaß der Informationssuche zur Abwägung von Risiko und Nutzen; Personen mit hohen Werten in diesem Bereich haben oft eine geringere Impfbereitschaft, da sie durch Falschwissen das Risiko der Impfung höher einschätzen als den Nutzen und
5. gesellschaftliche Verantwortung (Collective Responsibility) beschreibt die prosoziale Motivation, schwächere Mitglieder der Gesellschaft durch eine Impfung zu schützen und erhöht die Impfbereitschaft [8].

Welches dieser Konstrukte eine zentrale Rolle spielt, ist abhängig vom Impfstoff. Studien zeigen, dass für das Pneumokokken-Impfverhalten Vertrauen und Risikowahrnehmung zentrale Faktoren sind [9]. Für die COVID-19-Impfintention hingegen zeigen Studien aus Großbritannien, den Niederlanden, Belgien und Portugal Vertrauen und gesellschaftliche Verantwortung als starke Prädiktoren [10–12]. Bei der Influenza-Impfung wurde das Impfverhalten vor allem durch Vertrauen und Risikowahrnehmung beeinflusst [13].

### Weitere Einflussfaktoren auf die Impfintention

Neben psychologischen Gründen des (Nicht‑)Impfens wirkt sich auch das Wissen über ein Krankheitsbild und die verfügbare Impfung auf die Impfintention aus [14]. Personen, die gut über die Risiken und Vorteile einer Impfung informiert sind, zeigen eine höhere Impfbereitschaft [15, 16]. Studien bestätigen, dass unzureichendes Wissen über Pneumokokken die Impfbereitschaft senkt [14, 17]. Auch bezogen auf Influenza führt unzureichendes Wissen über die Effektivität von Impfungen zu einer geringeren Impfbereitschaft [13]. Ein fundiertes Verständnis der Krankheit und ihrer Prävention ist daher relevant, um eine positive Impfentscheidung zu treffen. Ein Mangel an Wissen kann dagegen zu Misstrauen und infolgedessen zu Impfablehnung führen, da Vertrauen und Wissen eng verknüpft sind [17, 18].

In der Literatur wird mit zunehmendem Alter eine steigende Pneumokokken-Impfquote beobachtet, die jedoch ab einem Alter von 90 Jahren wieder sinkt [4]. Dies weist darauf hin, dass das Alter und/oder altersassoziierte Faktoren einen Einfluss auf die Impfentscheidung bei Pneumokokken haben. Der Einfluss des Geschlechts auf die Impfentscheidung ist nicht eindeutig belegt. Während einige Studien eine höhere Impfquote bei Frauen berichten, weisen andere auf eine erhöhte Impfbereitschaft bei Männern hin oder zeigen keine signifikanten geschlechtsspezifischen Unterschiede [17, 19, 20]. In Bayern war 2021 bei Frauen (36,3 %) über 60 Jahren eine deutlich höhere Impfquote zu beobachten als bei Männern (19,7 %; [4]). Als robuster Prädiktor für die Entscheidung für eine Pneumokokken-Impfung zeigt sich die Entscheidung für eine Influenza-Impfung in der vergangenen Impfsaison. Diese erhöht signifikant die Wahrscheinlichkeit der Akzeptanz der Pneumokokken-Impfung [21–23].

### Die Theory of Planned Behavior

Die Theory of Planned Behavior (TPB) ist ein psychologisches Modell, welches zur Erklärung und Vorhersage von individuellem Gesundheitsverhalten genutzt werden kann. Es erklärt Gesundheitsverhalten durch die Intention, ein Verhalten auszuführen. Die Intention wird beeinflusst von Einstellungen, subjektiven Normen und wahrgenommener Verhaltenskontrolle [24]. Die Intention ist gemäß dem Modell dabei der stärkste Prädiktor für tatsächliches Verhalten und stellt die bewusste Entscheidung dar, ein Verhalten auszuführen [24]. Studien zeigen, dass das TPB-Modell erfolgreich zur Erklärung der Impfintention genutzt werden kann, insbesondere die Einflussfaktoren „Einstellungen“ und „subjektive Normen“ scheinen von besonderer Relevanz [25, 26]. Es kann durch andere Konstrukte, wie die 5C-Skala, ergänzt werden, um eine umfassendere Erklärung der Impfintention zu ermöglichen [13].

**Abb. 1:**
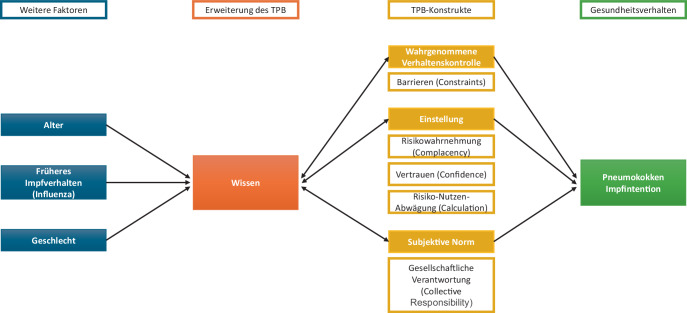
Das erweiterte Theory-of-Planned-Behavior-(TPB-)Modell. (Eigene Abbildung in Anlehnung an Ajzens TPB-Modell [24])

In dieser Arbeit wird ein Modell entwickelt, das die 5C-Skala in das TPB-Modell integriert. Dabei werden Vertrauen, Risikowahrnehmung und Risiko-Nutzen-Abwägung den Einstellungen im TPB-Modell zugeordnet (Abb. 1). Die subjektive Norm wird durch die wahrgenommene gesellschaftliche Verantwortung abgebildet und Barrieren beeinflussen die wahrgenommene Verhaltenskontrolle. Der Faktor „Wissen über Pneumokokken“ erweitert das Modell, indem er alle 3 TPB-Dimensionen beeinflusst: Wissen stärkt das Vertrauen, verbessert die Risikowahrnehmung und unterstützt die Risiko-Nutzen-Abwägung. Alter, Geschlecht und vorherige Impfungen, insbesondere die Influenza-Impfung, werden als modifizierende Variablen einbezogen, da sie die Impfintention beeinflussen können.

### Ziel und Forschungsfragen

Die vorliegende Studie untersucht die Impfintention in Bezug auf die Pneumokokken-Impfung bei Personen ab 60 Jahren in Deutschland. Das übergeordnete Ziel der Studie ist, ein besseres Verständnis für die Prädiktoren der Impfintention in dieser Altersgruppe zu entwickeln und so die Basis für die Entwicklung gezielter Interventionsstrategien zu bilden. Dabei wird das zuletzt vorgestellte, erweiterte TPB-Modell genutzt.

Die Studie untersucht folgende Fragestellungen:

1. Erhöhen Vertrauen in die Sicherheit der Pneumokokken-Impfung, ein hohes wahrgenommenes Risiko und eine hohe gesellschaftliche Verantwortung die Impfintention?
2. Senken wahrgenommene Barrieren und ein hohes Maß an Risiko-Nutzen-Abwägung die Impfintention?
3. Führt mehr Wissen über Pneumokokken-Erkrankungen zu einer höheren Impfintention?
4. Gibt es Zusammenhänge zwischen dem Alter, Geschlecht sowie einer vorherigen Influenza-Impfung und der Pneumokokken-Impfintention?

## Methode

### Datenerhebung

In der vorliegenden Studie wurden Querschnittsdaten einer Patient:innenbefragung im Innovationsfondsprojekt ALIVE – ALtersspezifische Impfinanspruchnahme VErbessern (Förderkennzeichen 01NVF20003) ausgewertet. Detaillierte Informationen zum Projekt können dem Studienprotokoll entnommen werden [27]. Von den 3 teilnehmenden regionalen KVen (Westfalen-Lippe, Nordrhein und Schleswig-Holstein) wurden Listen geeigneter Hausarztpraxen erstellt, die Personen ab 60 Jahren impfen. Diese Praxen wurden zufällig in Interventions- und Kontrollgruppe eingeteilt und die der Interventionsgruppe zur Teilnahme eingeladen. So konnten insgesamt 676 Hausärzt:innen für eine Projektteilnahme gewonnen werden. Ab April 2022 wurden in den Praxen Personen ab 60 Jahren rekrutiert und anonym per Fragebogen (papierbasiert) befragt, der zu Hause oder in der Praxis ausgefüllt werden konnte. Jede Praxis war verpflichtet, mindestens 25 Patient:innen für diese Befragungsrunde zu rekrutieren. Insgesamt haben 1117 Patient:innen den Fragebogen ausgefüllt und zurückgesendet.

### Erhebungsinstrumente

Impfintention stellt die abhängige Variable dar. Das zugehörige Item stammt aus der Studie von Betsch et al. [28] und lautete: „Stellen Sie sich vor, Sie wären gegen die folgenden Erkrankungen bzw. Erreger nicht geimpft. Wenn Sie nächste Woche die Möglichkeit hätten, sich gegen die folgenden Erkrankungen bzw. Erreger impfen zu lassen, wie würden Sie entscheiden?“ Die Antworten wurden auf einer Likert-Skala von 1 („auf keinen Fall impfen“) bis 7 („auf jeden Fall impfen“) erfasst.

Zur Erfassung der psychologischen Gründe des (Nicht‑)Impfens wurde eine Skala angelehnt an die Kurzform der impfbezogenen 5C-Skala nach Betsch et al. [7] genutzt, bei der die Risikowahrnehmung im Vergleich zur Originalskala umgepolt wurde. Die Kurzform umfasst 5 Fragen zu den 5 Konstrukten, beantwortet auf einer Skala von 1 („stimme keinesfalls zu“) bis 7 („stimme voll zu“; Tab. 1). Das Wissen über Pneumokokken-Erkrankungen und die Impfung wurde mit 4 Items erfasst, bewertet mit „Ja“, „Nein“ oder „weiß nicht“. Falsche Antworten (inkl. „weiß nicht“) wurden mit 0, richtige mit 1 kodiert, summiert und durch 4 geteilt, um einen Wissensindex von 0 bis 1 zu berechnen. Als weitere Kontrollvariablen wurden Alter und Geschlecht erfasst. Das Alter der Teilnehmenden wurde in Altersgruppen erfasst: 60–64, 65–69, 70–74, 75–79, 80–84 und 85 + Jahre. Diese Gruppen wurden von 1 bis 6 kodiert. Die Geschlechtsvariable wurde basierend auf der Frage: „Was ist Ihr Geschlecht?“, mit den Antwortoptionen „weiblich“, „männlich“ und „divers“ erhoben. Da „divers“ nicht gewählt wurde, erfolgte eine binäre Kodierung: 0 = „weiblich“, 1 = „männlich“. Früheres (Influenza‑)Impfverhalten wurde anhand der erhaltenen Impfungen in den letzten 12 Monaten erfasst (0 = nicht geimpft, 1 = geimpft).

**Tab. 1:** Operationalisierung von Impfverhalten und Wissen

Konstrukt	Item/Frage	Skala
Vertrauen	„Ich habe vollstes Vertrauen in die Sicherheit der Pneumokokken-Impfung“	1 = „stimme keinesfalls zu“, 7 = „stimme voll und ganz zu“
Risikowahrnehmung	„Ich fühle mich durch Krankheiten bedroht, die mit der Pneumokokken-Impfung verhindert werden können“	1 = „stimme keinesfalls zu“, 7 = „stimme voll und ganz zu“
Barrieren	„Alltagsstress hält mich davon ab, mich gegen Pneumokokken impfen zu lassen“	1 = „stimme keinesfalls zu“, 7 = „stimme voll und ganz zu“
Risiko-Nutzen-Abwägung	„Wenn ich darüber nachdenke, mich gegen Pneumokokken impfen zu lassen, wäge ich sorgfältig Nutzen und Risiken ab“	1 = „stimme keinesfalls zu“, 7 = „stimme voll und ganz zu“
Gesellschaftliche Verantwortung	„Wenn alle gegen Pneumokokken geimpft sind, brauche ich mich nicht auch noch impfen zu lassen“	1 = „stimme keinesfalls zu“, 7 = „stimme voll und ganz zu“
*Wissensskala*		*Skala: 0* *=* *falsch/weiß nicht, 1* *=* *richtig*
Wissen Item 1	„Pneumokokken können Lungenentzündungen auslösen“	0 = „falsch/weiß nicht“, 1 = „richtig“
Wissen Item 2	„Wer einmal eine Pneumokokken-Erkrankung durchgemacht hat, ist gegen Pneumokokken immun und muss nicht mehr geimpft werden“	0 = „falsch/weiß nicht“, 1 = „richtig“
Wissen Item 3	„Wenn man mit Pneumokokken in Berührung kommt, führt das immer zu Erkrankungen“	0 = „falsch/weiß nicht“, 1 = „richtig“
Wissen Item 4	„Pneumokokken können eine Sepsis (Blutvergiftung) auslösen“	0 = „falsch/weiß nicht“, 1 = „richtig“

Hinweis: Skala zum Impfverhalten ist angelehnt an die Kurzform der impfbezogenen 5C-Skala nach Betsch et al. [7]. Hierbei wurde das Item „Risikowahrnehmung“ umgepolt

### Datenauswertung

Die Variablen wahrgenommene Barrieren, Risiko-Nutzen-Abwägung und gesellschaftliche Verantwortung wurden umkodiert, sodass hohe Zustimmungswerte eine Ablehnung der entsprechenden Aussagen bedeuten. Dies erleichtert die Interpretation, da nun alle Variablen positiv mit der Impfintention korrelierten [8].

Fälle mit weniger als 50 % beantworteter Fragen im gesamten Fragebogen sowie solche ohne Angaben zur Pneumokokken-Impfintention wurden ausgeschlossen, sodass die Analysestichprobe 1000 Fälle betrug. Ausgeschlossene Personen waren zu 64,0 % weiblich und gehörten größtenteils den Altersgruppen der 70- bis 74-Jährigen (26,4 %) und 80- bis 84-Jährigen (22,7 %) an. Die Daten wurden deskriptiv ausgewertet und anschließend eine zweiseitige Korrelationsanalyse mittels Spearmans Rho (𝜌) durchgeführt, da dieser Koeffizient keine Normalverteilung voraussetzt und für ordinalskalierte Daten geeignet ist [29]. Zur Interpretation wurden Richtlinien nach Cohen [30] verwendet. Ein Signifikanzniveau von α = 0,05 wurde angewendet. Fehlende Werte wurden per „pairwise deletion“ ausgeschlossen und Ausreißer über 3 Standardabweichungen entfernt. Eine lineare multivariate Regressionsanalyse wurde mit der Methode der kleinsten Fehlerquadrate durchgeführt, um den Einfluss mehrerer unabhängiger Variablen auf die Impfintention zu untersuchen. Dabei wurde auf Multikollinearität getestet. Alle Analysen wurden mit IBM SPSS Statistics Version 29.0 durchgeführt [31].

## Ergebnisse

### Deskriptive Ergebnisse

Der Anteil weiblicher Patientinnen in der Befragung lag bei 59,1 %. Die am stärksten vertretene Altersgruppe war die der 60- bis 64-Jährigen mit 25,4 %, gefolgt von den 70- bis 74-Jährigen und 65- bis 69-Jährigen mit 20,9 % und 20,3 %. Die 60- bis 74-Jährigen machten somit 66,6 % der Stichprobe aus. Am seltensten vertreten waren über 85-Jährige mit 4,3 % (Tab. 2).

**Tab. 2:** Verteilung der Variablen

Variable	*n* (%)	MW/%	SD	Skala
Pneumokokken-Impfintention	1000 (100,0)	4,96	1,806	0–6
Vertrauen	981 (98,1)	5,62	1,405	1–7
Risikowahrnehmung	976 (97,6)	4,53	1,863	1–7
Barrieren	974 (97,4)	6,13	1,283	1–7
Risiko-Nutzen-Abwägung	980 (98,0)	3,47	1,887	1–7
Gesellschaftliche Verantwortung	977 (97,7)	6,21	1,224	1–7
Wissen	983 (983)	0,39	0,310	0–1
Influenza-Impfung	1000 (100,0)	77,0 %	–	0,1
Geschlecht (Referenz: weiblich)	996 (99,6)	40,4 %	–	0,1
Altersgruppe	978 (97,8)	–	–	0,1
*60–64 Jahre*	248 (25,4)	–	–	0,1
*65–69 Jahre*	199 (20,3)	–	–	0,1
*70–74 Jahre*	204 (20,9)	–	–	0,1
*75–79 Jahre*	151 (15,4)	–	–	0,1
*80–84 Jahre*	134 (13,7)	–	–	0,1
*85 Jahre und älter*	42 (4,3)	–	–	0,1

Hinweis: *n* = 1000, *MW* Mittelwert, *SD* Standardabweichung. Fälle mit weniger als 50 % beantworteter Fragen sowie solche ohne Angaben zur Pneumokokken-Impfintention wurden ausgeschlossen

Die große Mehrheit der Befragten gab an, in den letzten 12 Monaten vor dem Befragungszeitpunkt eine Impfung gegen Covid-19 (97,5 %) und gegen Influenza (77,0 %) erhalten zu haben. Seltener waren erwartungsgemäß die Impfungen gegen Herpes zoster, Pneumokokken und Tetanus, Diphtherie und/oder Keuchhusten, da diese nicht jährlich erfolgen. 1,1 % der Personen gaben an, Impfungen grundsätzlich abzulehnen.

Tab. 2 gibt einen Überblick über die Variablen. Die Pneumokokken-Impfintention zeigte einen Mittelwert von 4,92 (SD = 1,806) auf einer Skala von 0 bis 6, was eine eher hohe Impfintention widerspiegelt. Bei den Konstrukten der 5C-Skala lag der höchste Wert bei der gesellschaftlichen Verantwortung (Mean = 6,21, SD = 1,224), was auf eine starke prosoziale Motivation hinweist. Barrieren zeigten ebenfalls einen hohen Wert (Mean = 6,13, SD = 1,283), was für ein eher geringes Ausmaß wahrgenommener Hindernisse steht. Der Mittelwert der Variable Vertrauen in die Impfung lag bei 5,62 (SD = 1,405), was auf ein hohes Vertrauen hinweist, während das wahrgenommene Risiko (Mean = 4,53, SD = 1,863) eine mittlere Risikowahrnehmung anzeigt. Die Nutzen-Risiko-Abwägung (Mean = 3,47, SD = 1,887) zeigt, dass die Befragten ihr eigenes Abwägen von Risiken und Nutzen als moderat einschätzen.

Das Wissen über Pneumokokken (Index von 0 bis 1) ergab im Schnitt einen Wert von 0,39. Durchschnittlich wurden also 39 % der Fragen korrekt beantwortet. Die meisten Befragten wussten, dass Pneumokokken Lungenentzündungen auslösen können (68,3 %), während das Wissen über das Risiko einer Sepsis (26,4 %) und über die Übertragungswege von Pneumokokken (24,2 %) deutlich geringer ausfiel.

### Ergebnisse der Korrelationsanalyse

Die Korrelationsanalyse (Tab. 3) ergab signifikante positive Zusammenhänge von Vertrauen (𝜌 = 0,582), Risikowahrnehmung (𝜌 = 0,417), gesellschaftlicher Verantwortung (𝜌 = 0,292) und Barrieren (𝜌 = 0,273) auf die Impfintention (alle *p* < 0,01). Der Erhalt einer Influenza-Impfung zeigte ebenfalls einen signifikanten positiven Zusammenhang (𝜌 = 0,422, *p* < 0,01). Wissen über Pneumokokken korrelierte schwach positiv mit der Impfintention (𝜌 = 0,180, *p* < 0,01) und den meisten 5C-Komponenten. Ausnahme war die Risiko-Nutzen-Abwägung, die negativ korrelierte (𝜌 = −0,064, *p* < 0,05).

**Tab. 3:** Ergebnisse der Korrelationsanalyse, bivariate Spearman-Korrelation

*Variablen*		*1*	*2*	*3*	*4*	*5*	*6*	*7*	*8*	*9*	*10*
1	Intention Pneumokokken	–	–	–	–	–	–	–	–	–	–
2	Vertrauen	0,582^**^	–	–	–	–	–	–	–	–	–
3	Risikowahrnehmung	0,417^**^	0,517^**^	–	–	–	–	–	–	–	–
4	Barrieren	0,273^**^	0,358^**^	0,151^**^	–	–	–	–	–	–	–
5	Risiko-Nutzen-Abwägung	−0,022	−0,060	−0,120^**^	−0,033	–	–	–	–	–	–
6	Gesellschaftliche Verantwortung	0,292^**^	0,412^**^	0,208^**^	0,616^**^	−0,000	–	–	–	–	–
7	Wissen Pneumokokken	0,180^**^	0,236^**^	0,142^**^	0,152^**^	−0,064^*^	0,198^**^	–	–	–	–
8	Influenza-Impfung	0,422^**^	0,305^**^	0,277^**^	0,197^**^	0,044	0,202^**^	0,117^**^	–	–	–
9	Geschlecht (Referenz: weiblich)	−0,129^**^	−0,048	−0,042	−0,041	0,009	−0,033	−0,105^**^	−0,009	–	–
10	Altersgruppe	0,042	0,067^*^	0,122^**^	−0,021	−0,005	−0,067^**^	−0,077^**^	0,123^**^	0,049	–

^**^Korrelation signifikant auf 0,01 Level (2-seitig)

^*^Korrelation signifikant auf 0,05 Level (2-seitig)

### Ergebnisse der Regressionsanalyse

Die multivariate lineare Regressionsanalyse (Tab. 4) erklärte 48,8 % der Varianz der Impfintention (angepasstes R^2^ = 0,488) und war statistisch signifikant (F (9, 943) = 101,887, *p* < 0,001). Der stärkste positive Prädiktor war das Vertrauen in die Sicherheit der Impfung (𝛽 = 0,514, *p* < 0,001).

**Tab. 4:** Ergebnisse der multivariaten linearen Regressionsanalyse

Koeffizienten	b	SD	$\beta$	t	*p*-Wert	(95,0 % KI) UG	OG
(Konstanten)	0,109	0,278	–	0,391	0,696	−0,437	0,654
Vertrauen	0,660	0,038	0,514	17,540	< 0,001	0,587	0,734
Risikowahrnehmung	0,084	0,027	0,086	3,144	0,002	0,031	0,136
Barrieren	0,029	0,039	0,021	0,737	0,461	−0,048	0,106
Risiko-Nutzen-Abwägung	−0,008	0,023	−0,009	−0,377	0,706	−0,053	0,036
Gesellschaftliche Verantwortung	−0,014	0,042	−0,009	−0,325	0,745	−0,096	0,069
Wissen	−0,027	0,143	−0,005	−0,190	0,849	−0,307	0,253
Influenza-Impfung	1,108	0,110	0,258	10,117	< 0,001	0,893	1,323
Geschlecht (Referenz: weiblich)	−0,163	0,086	−0,044	−1,899	0,058	−0,331	0,005
Altersgruppe	−0,061	0,028	−0,051	−2,149	0,032	−0,117	−0,005

Angepasstes R^2^ = 0,488; *n* = 1000

*KI* Konfidenzintervall, *UG* Untergrenze, *OG* Obergrenze, *SD* Standardabweichung

Auch die Risikowahrnehmung (𝛽 = 0,086, *p* = 0,002) zeigte einen signifikanten positiven Zusammenhang, wobei eine höhere Risikowahrnehmung durch Pneumokokken mit einer gesteigerten Impfintention verbunden war. Der Erhalt einer Influenza-Impfung in den letzten 12 Monaten zeigte ebenfalls einen signifikanten positiven Effekt auf die Pneumokokken-Impfintention (𝛽 = 0,153, *p* < 0,001). Je älter die Altersgruppe, desto geringer die Impfintention (𝛽 = −0,051, *p* = 0,032).

Andere Variablen der 5C-Skala wie wahrgenommene Barrieren, Nutzen-Risiko-Abwägung und gesellschaftliche Verantwortung sowie das Wissen über Pneumokokken zeigten in der Regressionsanalyse keine signifikanten Zusammenhänge.

**Abb. 2:**
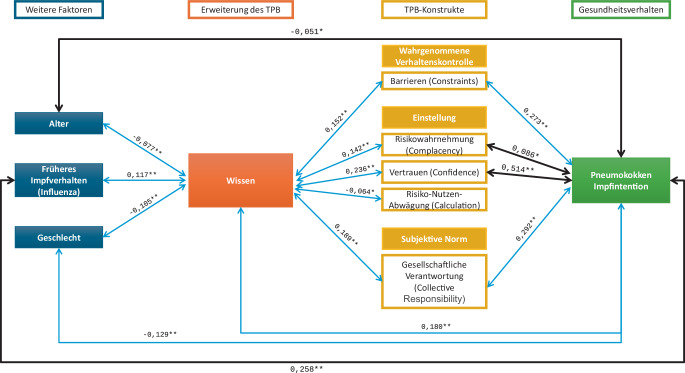
Integration der Ergebnisse in das erweiterte TPB-Modell, Hinweise: *schwarze Pfeile*: Ergebnisse der Regressionsanalyse, *β*-Koeffizient; *blaue Pfeile* Ergebnisse der Korrelationsanalyse, Spearman-Koeffizient, *2* *Sternchen*: Korrelation signifikant auf 0,01 Level. *Sternchen*: Korrelation signifikant auf 0,05 Level. (Eigene Abbildung)

Die Ergebnisse der Analysen sind in Abb. 2 zusammengefasst, wobei nur ausgewählte signifikante Zusammenhänge dargestellt werden.

## Diskussion

Pneumokokken sind die Hauptursache für schwere Atemwegsinfektionen und Todesfälle bei Infektionen der unteren Atemwege. Es wird empfohlen, verstärkt auch Maßnahmen für ältere Menschen zu ergreifen, da Präventionsinitiativen bisher meist auf Kinder unter 5 Jahren abzielen [1]. In der vorliegenden Studie wurden Prädiktoren der Pneumokokken-Impfintention von Personen ab 60 Jahren untersucht. Dabei erwiesen sich Vertrauen in die Sicherheit der Impfung und Risikowahrnehmung als zentral für die Pneumokokken-Impfintention. Dies steht im Einklang mit einer früheren Untersuchung zur Akzeptanz der Pneumokokken-Impfung von Personen ab 60 Jahren in Deutschland [9]. Für Influenza zeigten Vertrauen und Risikowahrnehmung den stärksten Effekt [13], während bei Covid-19 alle 5C-Konstrukte signifikant waren [10], jedoch in anderen Altersgruppen als hier untersucht. Diese Variabilität in den relevanten Faktoren je nach Impfstoff und Population unterstützt die Annahme, dass Impfmüdigkeit ein kontextabhängiges Phänomen ist [6]. Vertrauen in die Sicherheit der Impfung erweist sich oft als zentrales Element, um die Akzeptanz von Impfungen zu erhöhen [9, 11, 13]. Wenn Personen das Risiko, an einer Krankheit zu erkranken, als hoch einschätzen, kann dies ihr Vertrauen in die Notwendigkeit einer Impfung erhöhen. Dadurch nehmen sie den direkten Nutzen der Impfung stärker wahr, da sie diese als Schutz vor der Erkrankung sehen. Die gesellschaftliche Verantwortung als Motivation für Impfungen korreliert außerdem positiv mit dem Konstrukt Vertrauen. Dies legt nahe, dass Individuen ihre eigenen Gesundheitsentscheidungen als Teil eines größeren sozialen Kontexts betrachten. Wenn die eigene Rolle innerhalb der Gesellschaft bewusst ist und erkannt wird, dass die Impfung auch andere Personen schützt, kann dies das Vertrauen in die Impfung erhöhen. Denn Impfentscheidungen werden nicht nur auf individueller Ebene getroffen, sondern auch stark von sozialen Überzeugungen und Normen beeinflusst [32, 33].

Übereinstimmend mit den Ergebnissen früherer Studien [21, 23, 34] zeigte sich, dass die Bereitschaft zur Pneumokokken-Impfung höher war, wenn in der vorherigen Impfsaison bereits eine Influenza-Impfung in Anspruch genommen wurde. Ein Ansatz zur Erhöhung der Akzeptanz ist die gleichzeitige Verabreichung beider Impfungen, wobei die Pneumokokken-Impfung in der Regel nur alle 6 Jahre notwendig ist. Die Wirksamkeit dieser Strategie wurde bereits in einer Metaanalyse belegt [35]. Mit zunehmendem Alter war eine sinkende Impfbereitschaft zu beobachten; dies bestätigt auch eine Studie auf Basis von GKV-Daten, in der die Altersgruppe 90+ die geringste Impfquote aufwies [4]. Aus der Analyse geht nicht hervor, ob tatsächlich das Alter zugrunde zu legen ist oder ob der Effekt auf altersassoziierte Einschränkungen in der Mobilität und kognitiven Fähigkeiten zurückzuführen ist. Möglich wäre auch, dass sehr alte Menschen aufgrund gesundheitlicher Einschränkungen und Zugangsbarrieren oder einer geringeren Priorisierung von Präventionsmaßnahmen weniger oft eine Impfung in Anspruch nehmen [4]. Hier sollte weitergehend untersucht werden, wie ein steigendes Alter und die Nichtinanspruchnahme der Impfung zusammenhängen.

Das vorhandene Wissen über mit Pneumokokken assoziierte Erkrankungen und die Pneumokokken-Impfung zeigte in der Regressionsanalyse keinen signifikanten Zusammenhang mit der Impfintention.

Es ist bekannt, dass Empfehlungen von Ärzt:innen die Impfentscheidung beeinflussen können [13]. Dieser Aspekt wurde in der Studie nicht berücksichtigt, ist jedoch zentraler Bestandteil der komplexen Intervention des ALIVE-Projekts, aus dem die Daten stammen. Weitere Details finden sich im Studienprotokoll [27].

### Theory of Planned Behavior

In dieser Studie wurde die TPB als theoretische Basis genutzt und um die Variablen Wissen über Pneumokokken, Geschlecht, Alter und früheres Impfverhalten erweitert. Die TPB-Konstrukte wurden mittels der 5C-Skala erfasst. Die Ergebnisse zeigten eine Korrelation zwischen Wissen über Pneumokokken und den TPB-Konstrukten, was darauf hindeutet, dass ein verbessertes Verständnis der mit Pneumokokken assoziierten Erkrankungen und der Impfstoffwirkungen das Verhalten beeinflussen könnte. Obwohl Wissen über Pneumokokken keinen direkten Zusammenhang zur Impfintention in der Regressionsanalyse hatte, zeigte sich in der Korrelationsanalyse ein indirekter Einfluss über Variablen wie Risikowahrnehmung und Vertrauen, welche wiederum Prädiktoren der Impfintention darstellten. Wissen spielt somit eine indirekte Rolle, wie es auch bei der Covid-19-Impfung der Fall ist [36]. Um fundierte Schlussfolgerungen zu ziehen, sind weiterführende Untersuchungen unter Anwendung fortgeschrittener statistischer Methoden, die über einfache Korrelationsanalysen hinausgehen, erforderlich.

Die Verwendung der 5C-Skala ermöglichte eine detaillierte Erfassung der psychologischen Gründe des (Nicht‑)Impfens, welche die Impfentscheidungen beeinflussen. Besonders hervorzuheben sind das Vertrauen in die Impfung und die Risikowahrnehmung, die sich als starke Prädiktoren der Impfintention herausstellten und dem TPB-Konstrukt „Einstellung“ zugeordnet wurden. Diese Ergebnisse bestätigen frühere Untersuchungen, die zeigen, dass Einstellung der wichtigste Einflussfaktor für die Impfintention ist [37]. Das nicht alle TPB-Konstrukte die Impfintention erklären können, zeigt sich in Studien zur Influenza- und Pneumokokken-Impfung [25, 26].

### Limitationen und Stärken

Die Ergebnisse der Studie weisen folgende Limitationen auf: Zunächst können aufgrund der gewählten Methode und Datengrundlage keine kausalen Zusammenhänge abgeleitet werden. Um der Komplexität des erweiterten TPB-Modells gerecht zu werden, sind weiterführende statistische Analysen erforderlich, insbesondere zur tiefergehenden Untersuchung der bislang nur durch Korrelationsanalysen erfassten Zusammenhänge.

Ein weiterer potenzieller Schwachpunkt liegt im Selektionsbias, da die Rekrutierung der Teilnehmenden über Hausärzt:innen erfolgte. Folglich werden ausschließlich Personen erreicht, die eine/einen Hausärzt:in haben und diese/n auch aufsuchen und somit empfänglicher für Impfungen sind. Dies könnte außerdem dazu führen, dass Hausärzt:innen eher Patient:innen auswählen, die der Impfung positiv gegenüberstehen, und es könnte zu einer Überrepräsentation von Personen mit höherem Gesundheitsbewusstsein kommen, da diese vermutlich eher an solchen Befragungen teilnehmen. Ebenso machten Hochaltrige (über 85-Jährige) nur 4,3 % der Studienpopulation aus. Dadurch wird die Generalisierbarkeit der Ergebnisse eingeschränkt. Zudem könnte der Einfluss der Covid-19-Pandemie das Antwortverhalten beeinflusst haben, da das Bewusstsein für Impfungen in der breiten Öffentlichkeit gestiegen ist [38, 39]. Insbesondere bei den 5C-Konstrukten könnte die hohe Zustimmung auf pandemiebedingte Veränderungen im Impfverhalten zurückzuführen sein [40]. Eine methodische Schwäche betrifft die Intentions-Verhaltens-Lücke des TPB-Modells, da die Intention, sich impfen zu lassen, nicht notwendigerweise das tatsächliche Verhalten widerspiegelt [41]. Eine ebenfalls relevante Limitation der Studie besteht darin, dass neben Alter, Geschlecht und vorherigen Impfungen keine weiteren potenziell modifizierenden Faktoren berücksichtigt wurden, wie beispielsweise der funktionelle oder kognitive Status der Teilnehmenden, was die Aussagekraft der Ergebnisse einschränken könnte.

Eine wesentliche Stärke dieser Studie liegt in der Integration der 5C-Konstrukte in das TPB-Modell, welches eine solide theoretische Grundlage für die Untersuchung der Impfintention bietet. Die Anwendung einer etablierten Theorie ermöglicht ein umfassendes Verständnis der Einflussfaktoren auf die Impfintention. Positiv zu bewerten ist darüber hinaus die hohe erklärte Varianz durch die lineare Regression, da sie zeigt, dass das Modell einen großen Teil der Streuung der abhängigen Variable erfasst. Dies deutet auf eine gute Anpassung des Modells an die Daten hin und erhöht die Aussagekraft und Zuverlässigkeit der Ergebnisse. Eine weitere Stärke der Studie liegt zudem in der systematischen Rekrutierung der Hausärzt:innen über die KVen der beteiligten Regionen. Durch die Vorauswahl der Praxen wurde sichergestellt, dass nur Hausarztpraxen aufgenommen wurden, die eine ausreichend hohe Anzahl Impfungen bei älteren Versicherten durchführten. Dies, kombiniert mit der anschließenden Randomisierung, minimierte die Gefahr einer Selbstselektion und schuf eine solide Grundlage für die Generalisierbarkeit der Ergebnisse. Mit einem Frauenanteil von 59,1 % ist die Stichprobe außerdem in etwa repräsentativ für die über 65-jährige Bevölkerung in Deutschland, hier liegt der Frauenanteil bei 56 % [42]. Darüber hinaus wurde mit einer großen Stichprobe eine robuste Datenbasis geschaffen.

## Fazit

Die Ergebnisse zur 5C-Skala weisen darauf hin, dass insbesondere Vertrauen in die Impfung und eine gesteigerte Risikowahrnehmung die Impfintention positiv beeinflussen. Impfkampagnen, die das Vertrauen und die Risikowahrnehmung erhöhen, können daher potenziell die Impfintention steigern. Dies könnte z. B. durch Maßnahmen erreicht werden, die das Wissen über Pneumokokken, die durch sie ausgelösten Erkrankungen und potenzielle, gefährliche Folgen sowie über die Impfstoffwirkung erhöhen. Weitere Forschung ist erforderlich, um den genauen Einfluss von Wissen über Pneumokokken auf die Impfintention zu klären. Alter und Geschlecht spielten in dieser Analyse eine geringe Rolle, sollten jedoch weiter untersucht werden, um Hintergründe der Zusammenhänge besser zu verstehen und gezielte Interventionen zu ermöglichen. Auch sollten Faktoren wie sozioökonomischer Status und Migrationshintergrund beachtet werden, um alle Bevölkerungsgruppen zu erreichen. Maßnahmen zur Steigerung der Impfintention bei Personen ab 60 Jahren sollten auf einer soliden Datengrundlage basieren und regelmäßig evaluiert werden, um die Wirksamkeit zu gewährleisten und die Impfquoten in dieser Hochrisikogruppe nachhaltig zu erhöhen.
